# Global stratospheric methane loss from satellite observations

**DOI:** 10.1073/pnas.2529774123

**Published:** 2026-02-09

**Authors:** Qiang Fu, Cong Dong

**Affiliations:** ^a^Department of Atmospheric and Climate Science, University of Washington, Seattle, WA 98195

**Keywords:** methane budget, stratospheric chemical loss, global CH4 diabatic flux

## Abstract

Stratospheric CH_4_ oxidation represents both an important sink in the global CH_4_ budget and a major source of stratospheric water vapor and hydrogen radicals, exerting strong influences on global climate and ozone chemistry. Yet, the magnitude of stratospheric CH_4_ chemical loss remains highly uncertain, with previous estimates largely relying on chemistry-climate models (CCMs). Here, we present an observationally based estimate of stratospheric CH_4_ loss (*L_STR_*), derived from the CH_4_ diabatic flux across the isentropic surface fitted to the tropical tropopause, using satellite measurements of CH_4_ concentration, temperature, and radiative heating rates for 2007–2010. We obtain an *L_STR_* of 49.8 ± 7.8 Tg/y, compared with 38.1 Tg/y estimated from reanalysis, and 25.7 Tg/y (range: 19.6 to 35.9 Tg/y) derived from CCMs, indicating that both reanalysis and CCMs systematically underestimate stratospheric CH_4_ loss. We show that discrepancies in global CH_4_ diabatic fluxes from the reanalysis and CCMs, when compared with observations, are mainly driven by biases in CH_4_ concentrations and further enhanced by errors in temperature and radiative heating. Substituting our observational estimate for the model-based stratospheric loss in the bottom-up global CH_4_ budget reduces the reported imbalance for the 2000s from 23 to 3 Tg/y, bringing it into close agreement with the 5 Tg/y (range: −4 to 13 Tg/y) imbalance inferred from top-down estimates. These findings highlight the critical role of observational constraints on *L_STR_* in reconciling the global CH_4_ budget. They also carry important implications for understanding stratospheric water vapor and ozone chemistry.

## Significance

Methane (CH_4_) is the second most important human-driven greenhouse gas after carbon dioxide, but its variability and long-term increase are not well understood, partly because of large uncertainties in how it is removed from the atmosphere. One important removal pathway is CH_4_ oxidation in the stratosphere, which also produces water vapor and reactive chemicals that affect climate and ozone. Until now, estimates of the stratospheric CH_4_ loss have relied only on models. Using satellite observations, we provide an observationally based estimate, showing that models systematically underestimate this loss. Incorporating our result into the global methane budget greatly reduces existing imbalances, helping reconcile top-down and bottom-up estimates and improving confidence in methane-climate assessments.

Methane (CH_4_) is the second most important anthropogenic greenhouse gas after carbon dioxide in terms of climate forcing (1). However, the drivers of its variability and long-term growth remain poorly understood (2–7). The key challenges in attributing the observed atmospheric CH_4_ growth are the diversity of its sources, and the uncertain magnitude and temporal variability of its destruction by short-lived, highly variable hydroxyl radicals (OH). According to the latest global CH_4_ budget estimates for 2000–2009^*^ (8), total emissions were 543 Tg/y from top-down approaches and 638 Tg/y from bottom-up approaches, with corresponding sink estimates of 538 and 615 Tg/y. These values imply imbalances of 5 and 23 Tg/y, respectively. Because the rate of change in atmospheric CH_4_ is governed by this imbalance—the small difference between two large terms—even biases of 10 to 20 Tg/y in individual sources or sinks are significant (8).

In the stratosphere, the primary sink for CH_4_ is its reaction with OH, producing the methyl radical (CH_3_) and water vapor (H_2_O) (9). Additional loss processes involve reactions with excited oxygen atoms [O(^1^D)] and chlorine atoms (Cl), yielding CH_3_ and OH or HCl, respectively (10). Through these pathways, CH_4_ acts as a sink for reactive chlorine, underscoring its importance in stratospheric ozone chemistry (11, 12), while also serving as a major source of stratospheric water vapor and thereby influencing global climate (13–16).

The chemical loss of stratospheric CH_4_ remains highly uncertain (8, 17). Based on chemistry–climate model simulations contributed to CMIP6 activity, Saunois et al. (8) estimated a stratospheric CH_4_ sink of ~37 Tg/y above the 200 hPa level for 2000–2009, with a range of 27 to 51 Tg/y. Reducing this uncertainty is critical for advancing our understanding of stratospheric CH_4_ oxidation and its impacts on ozone chemistry, stratospheric water vapor, and the global CH_4_ budget. To date, however, all estimates of stratospheric CH_4_ loss are model-based, as no direct observational constraints are available. Given the observationally derived ratio of stratospheric H_2_O production to CH_4_ destruction (18), an observational estimate of stratospheric CH_4_ loss is also required to quantify the H_2_O production rate. This, in turn, allows us to isolate the contributions of other sinks and sources—such as tropical and midlatitude overshooting convection and polar stratospheric clouds—to the global stratospheric H_2_O budget, which remains poorly constrained (19–21).

**Fig. 1.**
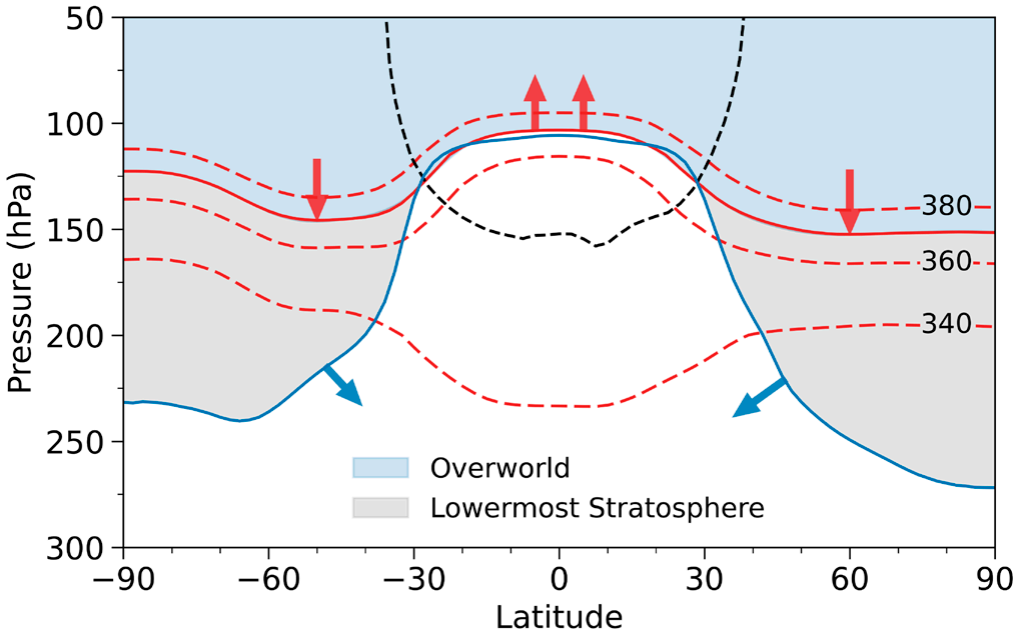
The stratospheric components considered in this study. The blue solid line is the WMO lapse-rate tropopause, above (below) which lies the stratosphere (troposphere). The black dashed line is the zero diabatic heating contour. The red solid line is the isentropic surface fitted to the tropical tropopause, equatorward of the latitudes with zero tropopause diabatic heating. The fitted isentrope serves as the upper boundary of the extratropical lowermost stratosphere (light-gray shading) and provides an approximate tropical tropopause. The light-blue shading above the fitted isentrope is the overworld of the stratosphere. Blue arrows indicate net tropopause fluxes in the extratropics, while downward and upward red arrows indicate diabatic fluxes across the fitted isentrope in the extratropics and tropics, respectively. Red dashed lines show the 340, 360, and 380 K isentropic surfaces.

Based on mass conservation, the rate of change of stratospheric CH_4_ mass above an isentropic surface can be expressed as:

[1] $$\frac{{\mathrm{dM}}_{{\mathrm{CH}}_{4}}}{\mathrm{dt}}=F_{d}^{{\mathrm{CH}}_{4}}-L+B,$$

where $M_{{\mathrm{CH}}_{4}}$ is the CH_4_ mass above a given isentropic surface, $F_{d}^{{\mathrm{CH}}_{4}}$ is the global CH_4_ diabatic flux across that surface, and *L* is the total CH_4_ loss rate within the overlying stratosphere. Isentropic surfaces can shift vertically in response to atmospheric temperature changes, and the overlying CH_4_ mass changes accordingly. *B* represents the rate of change in CH_4_ mass associated with this “breathing” effect of the isentropic surface. For annual-mean conditions, both $\frac{{\mathrm{dM}}_{{\mathrm{CH}}_{4}}}{\mathrm{dt}}$ and *B* are small, and *L* is largely dominated by $F_{d}^{{\mathrm{CH}}_{4}}$. We are particularly interested in applying Eq. **1** to the isentropic surface fitted to the tropical tropopause^†^, where *L* represents the global CH_4_ loss rate in the overworld—defined as the stratospheric region above this isentropic surface (Fig. 1) (22).

$F_{d}^{{\mathrm{CH}}_{4}}$ is determined from CH_4_ concentration, temperature, and radiative heating rates (*Materials and Methods*). Here we propose to observationally estimate *L* by deriving $F_{d}^{{\mathrm{CH}}_{4}}$ using satellite observations. Specifically, CH_4_ mixing ratios are taken from ACE-FTS (Atmospheric Chemistry Experiment–Fourier Transform Spectrometer) (23, 24), temperatures from COSMIC (Constellation Observing System for Meteorology, Ionosphere, and Climate) (25–27), and radiative heating rates from A-Train satellite measurements (28–30). $\frac{{\mathrm{dM}}_{{\mathrm{CH}}_{4}}}{\mathrm{dt}}$ and *B* in Eq. **1** can readily be derived from satellite CH_4_ and temperature observations. In addition to estimating the global overworld CH_4_ loss, we also quantify CH_4_ loss in the lowermost stratosphere—defined as the extratropical stratospheric region between the extratropical tropopause and the fitted isentropic surface (Fig. 1) (22)—as well as CH_4_ loss in the entire stratosphere. We further evaluate the CH_4_ exchange between the stratosphere and troposphere (Fig. 1). With the recent availability of a greenhouse gas reanalysis (31) providing CH_4_ concentrations, we also derived these quantities using the reanalysis CH_4_ along with ERA5 reanalysis temperature and radiative heating rates (32) for comparison. Furthermore, we derived these quantities from model simulations in both CCMI-1 (33) and CCMI-2 (34).

Based on observations for 2007–2010, the estimated CH_4_ losses are 47.1 ± 7.5 Tg/y in the overworld, 2.7 ± 0.7 Tg/y in the lowermost stratosphere, and 49.8 ± 7.8 Tg/y for the entire stratosphere above tropopause. The CH_4_ loss above 200 hPa is 57.1 ± 9.0 Tg/y, based on an empirical relationship with the overworld CH_4_ loss. Our results show that both reanalysis and models systematically underestimate stratospheric CH_4_ loss compared with observational estimates. Substituting our observationally based stratospheric loss for the model-derived mean used in Saunois et al. (8) largely reduces the imbalance in the global CH_4_ budget for the 2000 s from the bottom-up approach, bringing it into an agreement with the imbalance inferred from the top-down approach. This demonstrates that observational constraints on stratospheric CH_4_ loss play a key role in reconciling discrepancies between the two approaches.

## Stratospheric CH_4_ Budget

From Eq. **1**, the total stratospheric CH_4_ loss above an isentropic surface in the stratosphere can be written as:

[2] $$L=F_{d}^{{\mathrm{CH}}_{4}}-dMB,$$

where *dMB* $=\frac{{\mathrm{dM}}_{{\mathrm{CH}}_{4}}}{\mathrm{dt}}-B$. We first examine the budget terms in Eq. (**2**) using simulation outputs from the CCMI-2 refD1 experiment (34), produced with the GEOSCCM model by the NASA Goddard Space Flight Center (NASA-GSFC) modeling team (35, 36). Details of the calculations of $F_{d}^{{\mathrm{CH}}_{4}}$ and dMB are provided in *Materials and Methods*. Notably, GEOSCCM is the only CCMI-2 model that provides both CH_4_ loss and radiation heating rates.

**Fig. 2.**
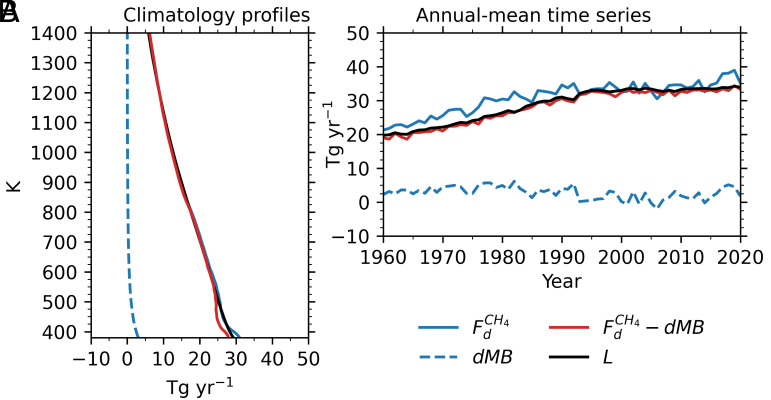
(*A*) Climatology profiles of global CH_4_ diabatic flux across different stratospheric isentropic surfaces ($F_{d}^{{\mathrm{CH}}_{4}}$, blue solid), the rate of change of CH_4_ mass above the isentropic levels with the “breath” effect of the isentropic surface removed (*dMB*, blue dashed), $F_{d}^{{\mathrm{CH}}_{4}}$ *– dMB* (red solid), and CH_4_ chemical loss above the isentropic levels (*L*, black solid), averaged over 1960–2020, from CCMI-2 GEOSCCM. (*B*) annual-mean time series of the same quantities as in (*A*) but evaluated for the isentropic surface fitted to the tropical tropopause.

Fig. 2*A* shows the vertical profiles of the budget terms in Eq. **2**, while Fig. 2*B* presents their annual-mean time series for the overworld with respect to the isentropic surface fitted to the tropical tropopause. As expected from Eq. **2**, *L* is well reproduced by $F_{d}^{{\mathrm{CH}}_{4}}-dMB$ throughout the atmosphere (Fig. 2*A*) and for all times (Fig. 2*B*). The total stratospheric CH_4_ loss above an isentropic surface is largely determined by the diabatic flux passing through the surface, with dMB contributing only minimally.

In this study, the CH_4_ diabatic flux and chemical loss with reference to the isentropic surface fitted to the tropical tropopause are noted as $F_{d,FI}^{{\mathrm{CH}}_{4}}$ and *L_OV_*, respectively. For this isentropic surface, GEOSCCM simulation averaged over 1960-2020 yields values of 28.8, 30.9, 2.8, and 28.1 Tg/y for *L_OV_,* $F_{d,FI}^{{\mathrm{CH}}_{4}}$, dMB, and $F_{d,FI}^{{\mathrm{CH}}_{4}}-dMB$, respectively (Fig. 2*B*). Thus, CH_4_ loss in the overworld can be well estimated by $F_{d,FI}^{{\mathrm{CH}}_{4}}-dMB$, and even by $F_{d,FI}^{{\mathrm{CH}}_{4}}$ alone, with small differences of −0.7 and 2.1 Tg/y, respectively.

The total CH_4_ loss in the stratosphere above the tropopause, denoted as *L_STR_*, is the sum of the loss in the overworld (*L_OV_*) and in the lowermost stratosphere (*L_LM_*) (Fig. 1):

[3] $$L_{\mathrm{STR}}=L_{\mathrm{OV}}+L_{\mathrm{LM}}.$$

While *L_OV_* can be obtained from Eq. **2** based on observations, *L_LM_* cannot. However, CCMI simulations show that *L_LM_* is relatively small (< 2.5 Tg/y) and strongly correlated with *L_OV_* (*SI Appendix*, Fig. S1). Therefore, *L_LM_* can be estimated from *L_OV_* using the empirical relationship shown in *SI Appendix*, Fig. S1, allowing *L_STR_* to be derived from *L_OV_*.

## Stratospheric CH_4_ Loss from Observations and Its Implications for the Global CH_4_ Budget

*L_OV_* can be obtained as $F_{d,FI}^{{\mathrm{CH}}_{4}}-dMB$ (Eq. **2** and Fig. 2). As both $F_{d,FI}^{{\mathrm{CH}}_{4}}$ and dMB can be derived from observations, the CH_4_ losses *L_OV_*, *L_LM_*, and *L_STR_* can likewise be estimated from $F_{d,FI}^{{\mathrm{CH}}_{4}}-dMB$ using observational data. While the relationship between *L_LM_* and *L_OV_* is based on CCMI model simulations (*SI Appendix*, Fig. S1), any bias from this model-derived relationship is expected to have little impact on *L_STR_*, as *L_LM_* is relatively small.

The isentropic surface aligned to tropical tropopause serves as the upper boundary of the lowermost stratosphere and the lower boundary of the overworld (Fig. 1). The diabatic flux across this surface is widely used in estimates of stratosphere–troposphere exchange (STE) for air mass and O_3_ (30, 37–42). Deriving $F_{d,FI}^{{\mathrm{CH}}_{4}}$ requires profiles of radiative heating rates, temperature, and CH_4_ concentrations, with the latter also used to calculate *dMB*.

In this study, we use CH_4_ mixing ratios from ACE-FTS (23, 24), temperatures from COSMIC (25–27), and radiative heating rates from A-Train measurements (28–30). The observationally derived CH_4_ losses—*L_OV_*, *L_LM_*, and *L_STR_*—averaged over 2007–2010 are given in Table 1. The analysis is confined to this period due to the availability of A-Train radiative heating rate data. Uncertainties are estimated by accounting for observational errors in CH_4_ concentrations, temperature, and radiative heating rates, as well as uncertainties associated with the *L_LM_*–*L_OV_* relationship (*Materials and Methods*). Table 1 presents an observationally derived CH_4_ losses in the overworld, lowermost stratosphere, and the entire stratosphere, with values of 47.1 ± 7.5, 2.7 ± 0.7, and 49.8 ± 7.8 Tg/y, respectively, averaged over 2007–2010. The uncertainties in *L_OV_* and *L_STR_* are primarily driven by errors in diabatic heating and CH_4_ concentrations, whereas the uncertainty in *L_LM_* arises from both observational errors and uncertainties in the *L_LM_*–*L_OV_* relationship (*Materials and Methods*).

**Table 1.** Global stratospheric CH_4_ loss (unit: Tg/y) based on observations, reanalysis, and multimodel mean (MMM) of CCMI-2 simulations for 2007–2010, shown for the overworld (*L_OV_*), lowermost stratosphere (*L_LM_*), the stratosphere above the WMO tropopause (*L_STR_*), and the stratosphere above 200 hPa (*L_200hPa_*)

	*L_OV_*	*L_LM_*	*L_STR_*	*L_200hPa_*
Obs.	47.1 ± 7.5	2.7 ± 0.7	49.8 ± 7.8	57.1 ± 9.0
Reanalysis	35.9	2.1	38.1	43.7
MMM*	24.1 (18.4, 33.7)	1.5 (1.2, 2.2)	25.7 (19.6, 35.9)	29.6 (22.6, 42.0)

^*^Seven CCMI-2 models are used; among them, only GEOSCCM provides both radiative heating rates and CH_4_ chemical loss. For GEOSCCM, the simulated CH_4_ chemical loss is used, which shows only minor differences from the derived values based on Eq. **2** (Fig. 2).

Observational uncertainties are shown as ±2σ range, and model ranges are given in parentheses.

With the recent availability of a greenhouse gas reanalysis (31) providing CH_4_ concentrations, we also estimated stratospheric CH_4_ loss using reanalysis CH_4_ together with ERA5 temperature and radiative heating rates (32), yielding an *L_STR_* of 38.1 Tg/y (Table 1). Since CH_4_ chemical loss is not included in the reanalysis, Table 1 thus also presents an estimate of stratospheric CH_4_ loss derived from such data based on Eqs. **2** and **3**. Table 1 further reports stratospheric CH_4_ loss from CCMI-2 models^‡^ (with CCMI-1 results given in *SI Appendix*, Table S1), yielding an *L_STR_* of 25.7 (range: 19.6 to 35.9) Tg/y. Thus, the observationally derived *L_STR_* exceeds the reanalysis-based estimate, which in turn is greater than that from CCMI models.

In Saunois et al.(8), stratospheric CH_4_ chemical loss is estimated from six chemistry–climate models participating in CMIP6. Using a tropopause pressure of 200 hPa, the simulations yield a mean estimate of 34 Tg/y for 2000–2009, with a range of 10 to 51 Tg/y. After excluding the lowest outlier—which implies an unrealistic stratospheric CH_4_ lifetime of several hundred years—Saunois et al. (8) report a mean of 37 Tg/y and a range of 27 to 51 Tg/y.

To compare our observational analysis with the stratospheric CH_4_ loss estimates of Saunois et al. (8), we first establish an empirical relationship between CH_4_ loss above 200 hPa (*L_200hPa_*) and in the overworld (*L_OV_*) using CCMI simulations. The relationship is *L_200hPa_* = 1.2*L_OV_* + 0.5, with a correlation coefficient of 1.0 (*SI Appendix*, Fig. S2), yielding an observationally derived *L_200hPa_* of 57.1 ± 9.0 Tg/y (Table 1). This estimate exceeds all model-simulated *L_200hPa_* reported in Saunois et al. (8). Substituting 57.1 Tg/y for their stratospheric loss of 37 Tg/y reduces the 2000–2009 imbalance from 23 to 3 Tg/y, consistent with their top-down imbalance estimates of 5 Tg/y (range: −4 to 13).

## Stratospheric CH_4_ Fluxes from Observations, Reanalysis, and CCMs

Because CH_4_ loss in the overworld (*L_OV_*) is primarily controlled by the CH_4_ diabatic flux across the isentropic surface fitted to the tropical tropopause (Fig. 2), and since both *L_LM_* and *L_200hPa_* are well determined by *L_OV_* (*SI Appendix*, Figs. S1 and S2), we focus on this flux as derived from observations, reanalysis, and CCMI simulations. We also investigate the factors contributing to the differences between reanalysis- and CCMI-based estimates and those from observations.

**Fig. 3.**
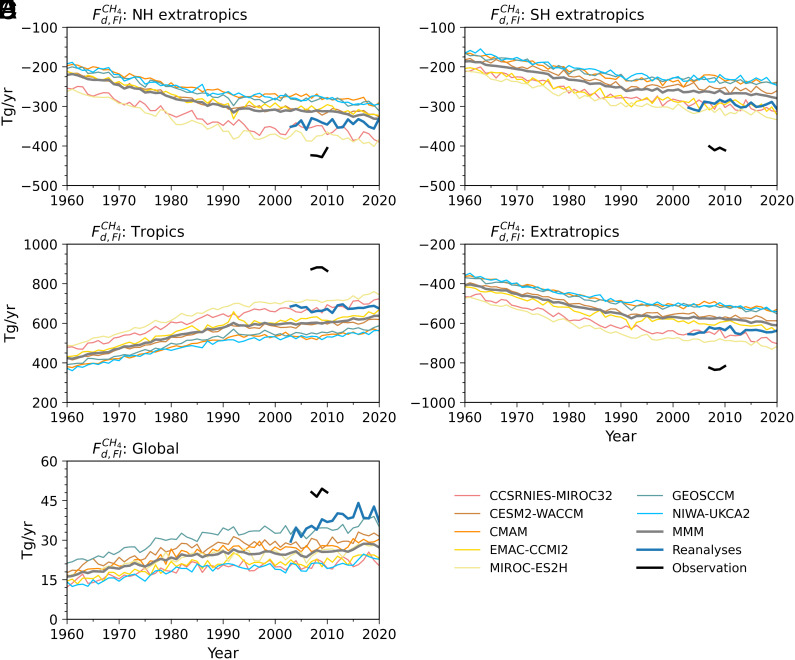
Time series of annual-mean CH_4_ diabatic flux across the isentropic surface fitted to the tropical tropopause ($F_{d,FI}^{{\mathrm{CH}}_{4}}$) over (*A*) NH extratropics, (*B*) SH extratropics, (*C*) tropics, (*D*) combined extratropics, and (*E*) globe. Thin colored lines represent individual CCMI-2 model results from 1960 to 2020; thick gray lines denote the multimodel mean (MMM). Thick blue lines indicate reanalysis results from 2003 to 2020, and thick black lines mark observational estimates from 2007 to 2010.

Fig. 3 shows the annual-mean time series of diabatic CH_4_ flux across the fitted isentropic surface for a) the Northern Hemisphere (NH) extratropics, b) the Southern Hemisphere (SH) extratropics, c) the tropics, d) the combined extratropics (NH+SH), and e) the globe (tropics + extratropics). Table 2 provides the corresponding averages over 2007–2010. Model simulations (1960–2020) show rapid increases in magnitudes from 1960 to 1990, followed by a much flatter trend thereafter. By contrast, reanalysis (2003–2020) indicates a significant increase in the global flux (Fig. 3*E*). The trends in both model simulations and reanalysis are dominated by changes in CH_4_ concentrations, a feature also noted in earlier work for O_3_ (40, 41). Observationally derived flux magnitudes are systematically larger than both reanalysis and model simulations; reanalysis magnitudes are larger than the multimodel mean (MMM) and lie near the upper bound of individual models. Flux magnitudes are greater over the NH extratropics than the SH extratropics, though the observed hemispheric difference is much smaller (Table 2). Over the tropics, fluxes from observations, reanalysis, and the MMM are 875.6 ± 132.7, 663.1, and 602.2 Tg/y, respectively; over the extratropics, they are –827.5 ± 127.7, –627.0, and –576.6 Tg/y; and over the globe, they are 48.1 ± 7.5, 36.0,^§^ and 25.6 Tg/y. CCMI-1 simulations yield results similar to those from CCMI-2 (*SI Appendix*, Fig. S3 and Table S2).

**Table 2.** (A) Observations, (B) reanalysis, and (C) MMM of CCMI-2 simulations over 2007–2010 for CH_4_ diabatic flux across the isentropic surface fitted to the tropical tropopause ($F_{d,FI}^{{\mathrm{CH}}_{4}}$), rate of change of CH_4_ mass in the lowermost stratosphere (*dM_CH4,LM_/dt*), chemical loss of CH_4_ in the lowermost stratosphere (*L_LM_*), and net CH_4_ flux across the tropopause (i.e., stratosphere–troposphere exchange, $F_{\mathrm{trop}}^{{\mathrm{CH}}_{4}}$)

	NH extratropics	SH extratropics	Tropics	Extratropics	Global
**(A) Obs.**					
$F_{d,FI}^{{\mathrm{CH}}_{4}}$	−420.6 ± 56.6	−406.9 ± 72.0	875.6 ± 132.7	−827.5 ± 127.7	48.1 ± 7.5
*dM_CH4,LM_/dt*	1.5 ± 0.5	1.4 ± 0.5	0.0 ± 0.0	2.9 ± 0.4	2.9 ± 0.4
*L_LM_*	1.1 ± 0.4	1.6 ± 0.4	0.0 ± 0.0	2.7 ± 0.7	2.7 ± 0.7
$F_{\mathrm{trop}}^{{\mathrm{CH}}_{4}}$	−418.0 ± 56.6	−404.0 ± 72.0	875.6 ± 132.7	−822.0 ± 127.7	53.6 ± 7.5
**(B) Reanalyses**					
$F_{d,FI}^{{\mathrm{CH}}_{4}}$	−337.9	−289.2	663.1	−627.0	36.0
*dM_CH4,LM_/dt*	1.0	−0.4	0.0	0.6	0.6
*L_LM_*	0.9	1.2	0.0	2.1	2.1
$F_{\mathrm{trop}}^{{\mathrm{CH}}_{4}}$	−335.9	−288.4	663.1	−624.3	38.7
**(C) MMM**					
$F_{d,FI}^{{\mathrm{CH}}_{4}}$	−312.1 (−375.4, −276.3)	−264.5 (−311.8, −226.3)	602.2 (528.7, 712.6)	−576.6 (−687.1, −503.5)	25.6 (20.7, 34.0)
*dM_CH4,LM_/dt*	0.2 (−1.6, 1.7)	0.7 (−0.5, 2.6)	0.0	0.9 (−1.4, 4.3)	0.9 (−1.4, 4.3)
*L_LM_*	0.7 (0.6, 1.0)	0.8 (0.6, 1.2)	0.0	1.5 (1.2, 2.2)	1.5 (1.2, 2.2)
$F_{\mathrm{trop}}^{{\mathrm{CH}}_{4}}$	−311.2 (−373.0, −274.0)	−263.0 (−308.3, −224.4)	602.2 (528.7, 712.6)	−574.2 (−681.4, −499.8)	28.0 (21.6, 36.2)

Results are shown for the NH extratropics, SH extratropics, tropics, the combined extratropics, and the globe. Observational uncertainties are shown as ±2σ range, and model ranges are given in parentheses. Units are Tg/y.

**Fig. 4.**
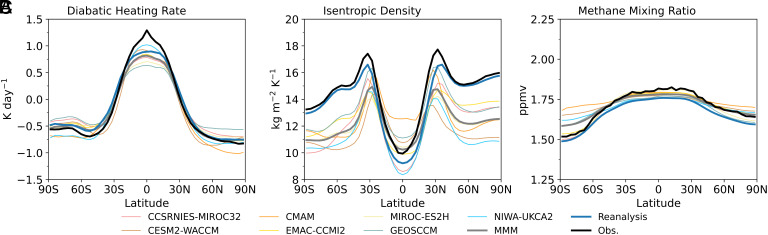
Latitudinal distributions of annual-mean zonal-mean (*A*) diabatic heating, (*B*) isentropic density, and (*C*) CH_4_ mixing ratio at the isentropic surface fitted to the tropical tropopause, averaged over 2007–2010, from individual CCMI-2 models (thin colored), the MMM (thick gray), reanalysis (thick blue), and observations (thick black).

The CH_4_ diabatic flux at isentropic surface is determined from three components: the diabatic heating, isentropic density (which depends on temperature), and the CH_4_ mixing ratios (*Materials and Methods*). Fig. 4 compares the latitudinal distributions of these fields from reanalysis, CCMI-2 and observations. Reanalysis diabatic heating magnitudes are generally weaker than observations, consistent with Wang and Fu (30), implying a weaker Brewer–Dobson circulation. This underestimate partly reflects the absence of thin cirrus clouds in reanalysis, which contribute additional heating (30, 42). Reanalysis isentropic densities and CH_4_ concentrations are systematically smaller than observations across all latitudes. Although CCMI-2 results exhibit large spreads, they consistently show weaker radiative heating in the tropics, lower isentropic densities in the extratropics, and smaller CH_4_ concentrations at low latitudes relative to observations (Fig. 4). Similar patterns are seen in CCMI-1, though CH_4_ concentrations are lower across all latitudes except at high SH latitudes (*SI Appendix*, Fig. S4).

To quantify the contribution of each variable’s deviation from observations to flux differences, we sequentially replace reanalysis/CCMI fields with observations following Wang and Fu (30). For example, we first replace diabatic heating (while retaining reanalysis/CCMI isentropic density and CH_4_), then replace isentropic density (still retaining reanalysis/CCMI CH_4_), and finally replace CH_4_, yielding a full observational set of fields. Flux differences between successive steps are attributed to the variable replaced at that step. While the ordering has only a minor effect, we evaluate all six possible replacement orders and report the average contribution associated with each variable.

**Fig. 5.**
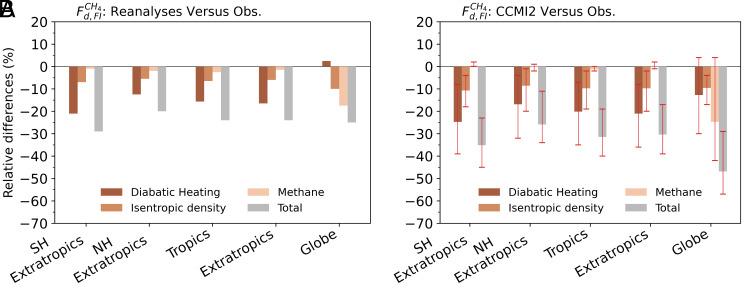
Relative differences (%) in CH_4_ diabatic flux ($F_{d,FI}^{{\mathrm{CH}}_{4}}$) across the fitted isentropic surface, attributed to differences in diabatic heating, isentropic density, and methane concentration, shown for (*A*) reanalysis versus observations and (*B*) CCMI-2 models versus observations. Results are shown for the SH extratropics, NH extratropics, tropics, combined extratropics, and globe. Bars in (*B*) show the MMM results while red error bars denote the range across individual models.

Fig. 5*A* shows that reanalysis underestimates diabatic fluxes by 20–29% across various regions. In the tropics, the SH and NH extratropics, and the combined extratropics, these differences arise mainly from diabatic heating (–16 to –21%), followed by isentropic density (–6 to –7%) and CH_4_ (–1 to –2%). Note that, across the tropics (~30°N/S), the impact of the substantial diabatic-heating underestimate in the reanalysis within ~ 20°N/S on the diabatic flux is partially offset by the impact of a modest overestimate at tropical latitudes outside this band (Fig. 4*A*). For the global diabatic flux—relevant to global stratospheric CH_4_ loss—the relative difference is –25%, dominated by CH_4_ (–18%), with smaller contributions from isentropic density (–10%) and diabatic heating (+2%).

Fig. 5*B* shows that CCMI-2 MMM underestimates diabatic fluxes by 26 to 47% across various regions. In the tropics, SH and NH extratropics, and combined extratropics, the differences arise mainly from diabatic heating (–17 to –25%), followed by isentropic density (–9 to –11%) and CH_4_ (–1 to 0%). For the global diabatic flux, the relative difference is –47%, dominated by CH_4_ (–25%), with smaller contributions from diabatic heating (–13%) and isentropic density (–10%). Similar results are found in CCMI-1 (*SI Appendix*, Fig. S5), though with larger discrepancies; for example, the global diabatic flux from the CCMI-1 MMM is 55% lower than observations.

The transition from a minor CH_4_ role regionally to a dominant one globally (Fig. 5 and *SI Appendix*, Fig. S5) reflects the fact that the global flux is a small residual of large opposing tropical and extratropical contributions, where diabatic heating effects largely cancel. It should be noted that this does not imply that the Brewer–Dobson circulation is unimportant, which strongly shapes the stratospheric CH_4_ distribution. These results highlight the need for accurate simulations and observations of stratospheric CH_4_, temperature, and radiative heating to obtain reliable estimates of stratospheric CH_4_ loss.

It is informative to examine the ratio of overworld CH_4_ loss to the CH_4_ flux entering the stratosphere—that is, *L_OV_* divided by $F_{d,FI}^{{\mathrm{CH}}_{4}}$ over the tropics. This overworld CH_4_ loss ratio is 5.4, 5.4, and 4% (range: 2.9 to 6.1%) for the observations, reanalysis, and CCMI-2, respectively. The identical ratios in the observations and reanalysis indicate that their difference in *L_OV_* is largely caused by differences in the amount of CH_4_ transported into the stratosphere. In contrast, the different ratio in the CCMI-2 simulations suggests that the discrepancies in *L_OV_* relative to observations arise from differences in both the incoming CH_4_ flux and the stratospheric CH_4_ chemistry.

## Stratosphere–Troposphere Exchange of CH_4_

The stratosphere–troposphere exchange (STE) of CH_4_ refers to the net flux of CH_4_ across the tropopause ($F_{\mathrm{trop}}^{{\mathrm{CH}}_{4}}$). Patra et al. (43) demonstrated that the modeled CH_4_ budget is highly sensitive to STE, indicating that simulated surface concentration growth rates are closely tied to CH_4_ exchange across the tropopause. More recently, Zhang et al. (7) showed that interannual variability in CH_4_ STE accounts for ~20% of the global mean variability in surface CH_4_ growth rates, with much larger impacts at high latitudes—explaining up to 80% of observed anomalies in Antarctica and 44% in the Arctic. In addition, analogous to Eq. **3**, we can express *L_STR_* = $F_{\mathrm{trop}}^{{\mathrm{CH}}_{4}}-{\mathrm{dMB}}_{\mathrm{trop}},$ with the tropopause as a reference surface. In this framework, the CH_4_ flux across the tropopause (STE) is directly related to total stratospheric CH_4_ loss.

The lowermost stratosphere mass budget approach provides a robust method to quantify STE of air and chemical species over the NH extratropics, SH extratropics, tropics, and the globe, and has been widely applied to estimate air mass and ozone STE in past, present, and future climates (e.g., 40, 41). In this framework, STE—defined as positive for net upward flux—is given by the sum of *L_LM_*, the rate of change in CH_4_ mass within the lowermost stratosphere (*dM_CH4,LM_/dt*), and the diabatic flux across the isentropic surface fitted to the tropical tropopause ($F_{d,FI}^{{\mathrm{CH}}_{4}}$) (*Materials and Methods*). These terms are quantified in Table 2 using observations, reanalyses, and CCMI-2 simulations (with CCMI-1 results given in *SI Appendix*, Table S2). When *L_LM_* is unavailable, it is inferred from *L_OV_* (Table 1 and *SI Appendix*, Fig. S1). Table 2 shows that the downward net CH_4_ flux from the extratropical stratosphere to the troposphere is 822, 624, and 574 Tg/y in observations, reanalysis, and the CCMI-2 MMM, respectively—values comparable to, or even larger than, the total tropospheric CH_4_ source (8). This flux, as derived from observations, can provide an important additional constraint on model simulations of the tropospheric CH_4_ budget.

Tables 1 and 2 show that the global net CH_4_ flux across the tropopause (STE) exceeds *L_STR_* by 3.8, 0.6, and 2.3 Tg/y in observations, reanalysis, and the CCMI-2 MMM, respectively.

## Discussions and Conclusions

While CH_4_ is the second most important anthropogenic greenhouse gas after CO_2_ in terms of radiative forcing, the drivers of its variability and long-term growth rate remain poorly understood. A central challenge in attributing the observed increase in atmospheric CH_4_ is the diversity of its emission sources and the large uncertainties in its primary sink—oxidation by short-lived, highly variable OH. Because the CH_4_ growth rate reflects the small imbalance between large source and sink terms, even small biases in either can have major implications.

In the stratosphere, CH_4_ oxidation plays multiple critical roles: it removes reactive chlorine, thereby influencing ozone chemistry, and it is a major source of stratospheric water vapor, which impacts climate. However, the magnitude of chemical CH_4_ loss in the stratosphere remains highly uncertain. To date, all estimates of stratospheric CH_4_ loss have been model-based, with no direct observational constraints available.

Here, we provide an observationally based estimate of stratospheric CH_4_ loss (*L_STR_*). *L_STR_* is determined from the global CH_4_ diabatic flux ($F_{d,FI}^{{\mathrm{CH}}_{4}}$) across the isentropic surface fitted to the tropical tropopause, the rate of change of CH_4_ mass above this surface ($\frac{{\mathrm{dM}}_{{\mathrm{CH}}_{4}}}{\mathrm{dt}}$), and the “breath” effect of the isentropic surface (*B*). The flux term $F_{d,FI}^{{\mathrm{CH}}_{4}}$ is derived from satellite observations of CH_4_ concentrations, temperature, and radiative heating rates, while $\frac{{\mathrm{dM}}_{{\mathrm{CH}}_{4}}}{\mathrm{dt}}$ and *B* are obtained from observed CH_4_ and temperature profiles. The contributions from $\frac{{\mathrm{dM}}_{{\mathrm{CH}}_{4}}}{\mathrm{dt}}$ and *B* are small. For the WMO tropopause, our results yield *L_STR_* values of 49.8 ± 7.8 Tg/y from observations, compared with 38.1 Tg/y from reanalysis and 25.7 Tg/y (range: 19.6 to 35.9 Tg/y) from CCMI-2 simulations. Applying the stratospheric H_2_O production-to-CH_4_ destruction ratio from Hurst et al. (18), these CH_4_ losses translate to stratospheric H_2_O productions of 112.0, 85.7, and 57.8 Tg/y, respectively. Our results indicate that both reanalysis and CCMI-2 systematically underestimate stratospheric CH_4_ loss. We show that discrepancies in global CH_4_ diabatic fluxes between the reanalysis/CCMI models and observations are primarily due to biases in CH_4_ concentrations, further compounded by temperature and radiative heating biases.

Regarding the global CH_4_ budget, the latest estimates for 2000–2009 report total emissions of 543 Tg/y (top-down) and 638 Tg/y (bottom-up), with corresponding sinks of 538 and 615 Tg/y, respectively (8). These values imply imbalances of 5 and 23 Tg/y. Saunois et al. (8) adopted a tropopause pressure of 200 hPa. An *L_STR_* of 49.8 Tg/y corresponds to a CH_4_ loss above 200 hPa (*L_200hPa_*) of 57.1 Tg/y (Table 1). Replacing the model-based stratospheric loss used in the bottom-up budget with our observational estimate reduces its imbalance from 23 to 3 Tg/y, bringing it into an agreement with the top-down estimate of 5 (−4 to 13) Tg/y. This demonstrates the key role of observational constraints on stratospheric CH_4_ loss in reconciling top-down and bottom-up global CH_4_ budget.

In the context of climate change mitigation, atmospheric CH_4_ presents both an opportunity and a challenge. The opportunity arises from its relatively short atmospheric lifetime of ~10 years and the availability of technological and agronomic strategies to reduce emissions, enabling effective short-term climate interventions (44). The challenge lies in accurately quantifying the global CH_4_ budget and its variability (8, 45). Our observationally based *L_STR_* estimates reduce a major source of bias and significantly enhance our understanding of the global CH_4_ budget. Furthermore, our findings have important implications for stratospheric water vapor, ozone chemistry, and the role of OH and circulation in the stratosphere.

## Materials and Methods

### Satellite Data.

We use CH_4_ mixing ratios from ACE-FTS (Atmospheric Chemistry Experiment–Fourier Transform Spectrometer) (23, 24, 46), temperatures from COSMIC (Constellation Observing System for Meteorology, Ionosphere, and Climate) (25–27), and radiative heating rates from A-Train satellite measurements (28–30).

For CH_4_, we use ACE-FTS version 5.2. In a validation study, De Maziere et al. (23) reported that ACE-FTS version 2.2 CH_4_ profiles had an accuracy within 10% in the upper troposphere and lower stratosphere. Although ACE-FTS CH_4_ data have not been formally validated since that study, retrievals generally improve with successive versions. Thus 10% can be considered an upper bound for version 5.2, and its accuracy is likely higher.

The COSMIC Global Navigation Satellite Systems (GNSS)-radio occultation (RO) temperature retrievals have high vertical resolutions and are accurate in the upper troposphere and lower stratosphere. The averaged GNSS-RO temperature profiles have very high accuracy (~0.1 K) in the upper troposphere and lower stratosphere (47,48, 49). Validation studies show that COSMIC temperature profiles are in good agreement with radiosonde measurements (25–27).

Radiative heating rates are derived from CloudSat Level 2B-FLXHR-LIDAR net fluxes. Shortwave radiative heating rates are then adjusted using the methodology described previously (28, 29), as adopted by Wang and Fu (30), to obtain daily mean shortwave radiative heating rates. Corrections are applied to mitigate the known biases in clear-sky shortwave fluxes from the 2BFL dataset compared to CERES observations (29, 30). For the longwave radiative heating rates, all-sky values are computed as an average of daytime and nighttime clear-sky radiative heating rates plus nighttime cloud radiative heating rates, which excludes the effect of “solar background noise” inherent in CALIPSO measurements (50). Cloud radiative heating rates are determined as the difference between all-sky and clear-sky rates. We focus on the period from January 2007 to December 2010 (30) because the 2BFL product is only available from July 7, 2006, to April 17, 2011.

All data used are monthly from January 2007 to December 2010 and have been regridded to a 4° × 5° latitude-longitude resolution. Because ACE-FTS provides relatively sparse spatial sampling, missing monthly observed CH_4_ values within 4° × 5° grid cells were filled as described in a later subsection.

### Reanalysis Data.

The Copernicus Atmosphere Monitoring Service (CAMS) at the European Centre for Medium-Range Weather Forecasts (ECMWF) has produced a greenhouse gas reanalysis for 2003–2020 (31, 51). This reanalysis integrates model output with satellite observations using ECMWF’s Integrated Forecasting System (IFS), providing a globally complete and consistent dataset for both CO_2_ and CH_4_. In addition, we use two meteorological reanalysis datasets: ERA5 from ECMWF (32) and MERRA-2 from NASA’s Modern-Era Retrospective Analysis for Research and Applications, Version 2 (52). Specifically, CH_4_ concentrations from CAMS, along with temperatures and radiative heating rates from ERA5, are employed for CH_4_ flux and mass calculations. Radiative heating rates from both ERA5 and MERRA-2 are used for uncertainty analysis.

### Model Data.

We use simulations from both phase 1 (CCMI-1) (33) and phase 2 (CCMI-2) (34) of the Chemistry-Climate Model Initiative (CCMI). Specifically, the CCMI-1 REF-C2 simulation covering 1960–2020 is analyzed, which applies historical forcings and observed sea surface conditions for 1960–2010, and follows the WMO (53) A1 scenario for ODS and the RCP6.0 pathway (54) for other GHGs, tropospheric ozone precursors, and aerosol and aerosol precursor emissions after 2010. The CCMI-2 REF-D2 simulation covering 1960–2020 is analyzed, which follows the specifications of the CMIP6 SSP2-4.5 scenario (55) with ODSs from WMO (56). The results from CCMI-2 simulations are presented in the main text, while those from CCMI-1 are provided in the Supplementary Information. Radiative heating rates are available from seven CCMI-2 models and nine CCMI-1 models. In addition, seven CCMI models (six from phase 1 and one from phase 2) provide CH_4_ chemical loss outputs, which are used to establish the relationships between *L_LM_* and *L_OV_* (*SI Appendix*, Fig. S1) and between *L_200hPa_* and *L_OV_* (*SI Appendix*, Fig. S2). Three of the seven models do not provide radiative heating rate output and are therefore excluded from the CH_4_ flux calculations. Outputs from seven CCMI-2 models are also used to assess the uncertainties associated with CH_4_ data gap in conjunction with the applied filling method.

### Global Stratospheric CH_4_ Budget Term Calculations.

The CH_4_ diabatic flux across an isentropic surface, $F_{d}^{{CH}_{4}}$, is calculated in the form (37)

[4] $$F_{d}^{CH_{4}}=\iint Q\sigma q_{CH_{4}}dA,$$

where $Q=R\frac{\theta }{T}$ is the diabatic heating rate, $\sigma =-g^{-1}\frac{\partial p}{\partial \theta }$ is the isentropic density, *q_CH4_* is the CH_4_ mass mixing ratio, *R* is the radiative heating rate, θ the potential temperature, *T* the temperature, *p* the pressure, *g* the gravitational acceleration, and *A* the area at the isentropic surface. For the global CH_4_ diabatic flux in Eq. **2**, the integration is carried out over the entire globe. Here upwelling flux is defined as positive.

The *dMB* term is defined as $\frac{{\mathrm{dM}}_{{\mathrm{CH}}_{4}}}{\mathrm{dt}}-B$, where $\frac{{\mathrm{dM}}_{{\mathrm{CH}}_{4}}}{\mathrm{dt}}$ is the rate of change of CH_4_ mass above the isentropic surface and *B* represents the “breath” effect associated with the temporal variation of that surface. To calculate $\frac{{\mathrm{dM}}_{{\mathrm{CH}}_{4}}}{\mathrm{dt}}$, we first compute the CH_4_ mass above the isentropic surface for each month and then take its temporal derivative. The *dMB* term is derived in the same way but using a climatological isentropic surface as the boundary, thereby removing the breath effect. Similar results were obtained when applying a climatological CH_4_ field together with a time-varying isentropic surface to compute *B* and subsequently obtain $\frac{{\mathrm{dM}}_{{\mathrm{CH}}_{4}}}{\mathrm{dt}}-B$.

### CH_4_ Stratosphere–Troposphere Exchange Based on Lowermost Stratosphere Mass Budget.

The lowermost stratosphere mass budget method, pioneered by Appenzeller et al. (37), has been widely used to quantify air mass and ozone STEs. Wang et al. (40) refined the method by introducing a dynamic isentropic upper boundary for the lowermost stratosphere (Fig. 1), determined monthly by minimizing the difference between an isentrope and the lapse-rate tropopause in the tropics (equatorward of the latitudes with zero diabatic heating). The fitted isentropic surface is then used to approximate the tropical tropopause, while the extratropical tropopause remains the lapse-rate definition. The zero diabatic heating latitude at the fitted isentrope defines the tropics–extratropics boundary: equatorward regions are tropical, those poleward are extratropical. The combined extratropical flux is the sum of the NH and SH extratropical fluxes, and the global flux is the sum of the tropical and extratropical fluxes.

The diabatic CH_4_ flux at the fitted isentropic surface, $F_{d,FI}^{{\mathrm{CH}}_{4}}$, is derived based on Eq. **4** by carrying out the integration over the fitted isentropic surface over the SH and NH extratropics and tropics. The net CH_4_ flux across the tropopause ($F_{\mathrm{trop}}^{{\mathrm{CH}}_{4}}$) in the NH/SH extratropics is the sum of $F_{d,FI}^{{\mathrm{CH}}_{4}}$, the lowermost stratospheric CH_4_ mass change rate ($\frac{dM_{{\mathrm{CH}}_{4},LM}}{\mathrm{dt}}$), and lowermost stratospheric CH_4_ chemical loss (*L_LM_*)

[5] $$F_{\mathrm{trop}}^{{\mathrm{CH}}_{4}}=F_{d,FI}^{{\mathrm{CH}}_{4}}+\frac{dM_{CH4,LM}}{\mathrm{dt}}+L_{\mathrm{LM}},$$

where the lowermost stratospheric CH_4_ mass, $M_{{\mathrm{CH}}_{4}}$, is

[6] $$M_{{\mathrm{CH}}_{4},LM}={∭}_{P_{i}}^{P_{t}}\frac{q_{{\mathrm{CH}}_{4}}\left(p\right)}{g}dpdA,$$

where *P_t_* and *P_i_* are pressures at the tropopause and fitted isentropic levels, respectively. In the tropics, $F_{\mathrm{trop}}^{{\mathrm{CH}}_{4}}=F_{d,FI}^{{\mathrm{CH}}_{4}}$.

### Filling Spatial Gaps in ACE-FTS CH_4_ Observations.

Missing ACE-FTS monthly CH_4_ observations were filled using the procedure described below:

1. All available Level-2 CH_4_ observations from 2007–2010 were assigned to the corresponding CAMS 0.75° × 0.75° grid cell according to their latitude, longitude, and month. For each grid cell and month, the mean of all available observations was calculated, while cells without observations were designated as missing.
2. Monthly differences between the CAMS reanalysis and the observations were computed for grid cells containing CH_4_ measurements during 2007–2010 and plotted as a function of latitude for each season (black dots in *SI Appendix*, Fig. S6).
3. For each 0.75° latitude band and season, monthly differences from all grid cells with observations were averaged over 2007–2010, yielding a seasonal zonal-mean climatology of reanalysis–observation differences.
4. Some latitude bands, particularly at high latitudes, contain missing values in the seasonal zonal-mean climatology. To achieve complete latitudinal coverage, missing values were filled by interpolation at mid- and low latitudes, and by nearest-neighbor values at high latitudes, producing a complete latitudinal coverage of the reanalysis-observation differences for each season (red lines in *SI Appendix*, Fig. S6).
5. Observational values were used for each month during 2007–2010 at grid cells wherever available. In grid cells without observations, missing values were filled using the reanalysis data adjusted by the corresponding seasonal zonal-mean climatology differences (i.e., the values for the season of the given month). The resulting fields were then interpolated onto a 4° × 5° latitude-longitude grid.

We used simulated CH_4_ fields from seven CCMI-2 models as pseudo-observations to evaluate the impact of the ACE-FTS data gaps, together with the filling method, on the derived $F_{d,FI}^{{\mathrm{CH}}_{4}}$ and *L_OV_*. Specifically, for each CCMI-2 model, CH_4_ fields were subsampled at the grid cells corresponding to ACE-FTS observation locations for each month during 2007-2010, with missing data filled using the method described above. $F_{d,FI}^{{\mathrm{CH}}_{4}}$ and *L_OV_* were then calculated and compared with those derived using the full model CH_4_ fields as a reference. As shown in *SI Appendix*, Fig. S7, the combined effects of missing data and the gap-filling procedure lead to differences in *L_OV_* of less than about 1 Tg/y, with a mean difference of 0.4 Tg/y, which is neglected in our uncertainty estimate.

### Observational Uncertainty Estimate.

The impact of observational uncertainties in CH_4_, temperature, and radiative heating rate is evaluated as follows. $F_{d,FI}^{{\mathrm{CH}}_{4}}$ is recalculated by (i) perturbing the CH_4_ concentration by ±10%, (ii) perturbing the temperature by ±0.5 K, and (iii) replacing the clear-sky radiative heating rate with that from ERA5 and MERRA2. Each perturbation yields two values, and the corresponding uncertainty *e* is taken as the larger of the absolute deviations. Assuming that the uncertainties in CH_4_, temperature, and radiative heating are independent, the total uncertainty is estimated as:

[7] $$e=\sqrt{{e^{2}}_{CH_{4}}+e_{\mathrm{Temp}}^{2}+e_{\mathrm{Rad}}^{2}}.$$

*SI Appendix*, Table S3 summaries the uncertainties in $F_{d,FI}^{{\mathrm{CH}}_{4}}$ arising from errors in CH_4_ concentrations, temperature, and diabatic heating. The uncertainty associated with diabatic heating exceeds that from CH_4_ except in the NH extratropics, while the temperature-related uncertainty is negligible. The uncertainty in *L_OV_* and its attribution is effectively the same as that in the global $F_{d,FI}^{{\mathrm{CH}}_{4}}$, since the impact of measurement errors on *L_OV_* through *dMB* is negligible.

The uncertainty in *L_LM_* arises from both observational uncertainties in *L_OV_* and the model-constructed relationship between *L_LM_* and *L_OV_*. The latter is quantified as twice the standard deviation of the differences between simulated *L_LM_* values and those from the fitted relationship (*SI Appendix*, Fig. S1). The uncertainties arising from the ACE-FTS CH_4_ data gaps, together with the filling method, are small (*SI Appendix*, Fig. S7).

## Supplementary Material

Appendix 01 (PDF)

## Data Availability

Observational satellite data used in this study are publicly available from ACE-FTS (https://databace.scisat.ca/level2/ace_v5.2/) (57), the COSMIC Data Analysis and Archive Center (https://cdaac-www.cosmic.ucar.edu/cdaac/) (58), and the CloudSat Data Processing Center (http://www.cloudsat.cira.colostate.edu/) (59). Reanalysis data were obtained from the Copernicus Atmosphere Monitoring Service for CAMS (https://ads.atmosphere.copernicus.eu/datasets/cams-global-reanalysis-eac4-monthly) (60), the ECMWF MARS archive for ERA5 (https://codes.ecmwf.int/grib/param-db/) (61), and the NASA Goddard Earth Sciences Data and Information Services Center for MERRA-2 (https://disc.gsfc.nasa.gov/datasets/M2TMNPRAD_5.12.4/summary) (62). Model simulation outputs from CCMI-1 and CCMI-2 are available from the Centre for Environmental Data Analysis (https://catalogue.ceda.ac.uk/uuid/9cc6b94df0f4469d8066d69b5df879d5 (63); https://catalogue.ceda.ac.uk/uuid/92dddf542adc44b5898f535be4179705) (64). All processed data and analysis software developed in this study are archived on Zenodo (https://zenodo.org/records/17335838) (65).

## Footnotes

^*^Saunois et al. present the global CH_4_ budget for 2000–2009, 2010–2019 and 2020 (8). We reference the 2000–2009 budget, as the atmospheric loss of CH_4_ is based on chemistry-climate model simulations driven by historical observed forcings.

^†^This study adopts the World Meteorological Organization (WMO) definition of the tropopause.

^‡^For CCMI models without explicit CH_4_ loss output, *L_STR_* is obtained using the same approach as for the observations, based on model CH_4_ concentrations, temperature, and radiative heating rates.

^§^The interannual variability (2σ) for the global CH_4_ flux in the reanalysis is 4.4 Tg/y.

## References

[1] Intergovernmental Panel on Climate Change (Ipcc), Climate Change 2021—The Physical Science Basis: Working Group I Contribution to the Sixth Assessment Report of the Intergovernmental Panel on Climate Change (Cambridge University Press, ed. 1, 2023).

[2] H. Schaefer , A 21st-century shift from fossil-fuel to biogenic methane emissions indicated by^13^CH_4_. Science **352**, 80–84 (2016). 26966190 10.1126/science.aad2705

[3] M. Rigby , Role of atmospheric oxidation in recent methane growth. Proc. Natl. Acad. Sci. **114**, 5373–5377 (2017). 28416657 10.1073/pnas.1616426114 PMC5448198

[4] A. J. Turner, C. Frankenberg, P. O. Wennberg, D. J. Jacob, Ambiguity in the causes for decadal trends in atmospheric methane and hydroxyl. Proc. Natl. Acad. Sci. **114**, 5367–5372 (2017). 28416668 10.1073/pnas.1616020114 PMC5448216

[5] S. Peng , Wetland emission and atmospheric sink changes explain methane growth in 2020. Nature **612**, 477–482 (2022). 36517714 10.1038/s41586-022-05447-w

[6] L. Feng, P. I. Palmer, R. J. Parker, M. F. Lunt, H. Bösch, Methane emissions are predominantly responsible for record-breaking atmospheric methane growth rates in 2020 and 2021. Atmos. Chem. Phys. **23**, 4863–4880 (2023).

[7] P. Zhang, Y. Zhang, R. Liang, W. Chen, X. Xie, Evaluation of the stratospheric contribution to the inter-annual variabilities of tropospheric methane growth rates. Geophys. Res. Lett. **50**, e2023GL103350 (2023).

[8] M. Saunois , Global methane budget 2000–2020. Earth Syst. Sci. Data **17**, 1873–1958 (2025).

[9] H. le Texier, S. Solomon, R. R. Garcia, The role of molecular hydrogen and methane oxidation in the water vapour budget of the stratosphere. Q. J. R. Meteorol. Soc. **114**, 281–295 (1988).

[10] G. P. Brasseur, S. Solomon, Aeronomy of the Middle Atmosphere: Chemistry and Physics of the Stratosphere and Mesosphere (Springer, Netherlands, 2005).

[11] R. W. Portmann, J. S. Daniel, A. R. Ravishankara, Stratospheric ozone depletion due to nitrous oxide: Influences of other gases. Philos. Trans. R. Soc. Lond. B Biol. Sci. **367**, 1256–1264 (2012). 22451111 10.1098/rstb.2011.0377 PMC3306630

[12] G. Zeng , Attribution of stratospheric and tropospheric ozone changes between 1850 and 2014 in CMIP6 models. J. Geophys. Res. Atmos. **127**, e2022JD036452 (2022).

[13] J. Lelieveld, P. J. Crutzen, Indirect chemical effects of methane on climate warming. Nature **355**, 339–342 (1992).

[14] J. Hansen , Efficacy of climate forcings. J. Geophys. Res.: Atmos. **110**, D18104 (2005). 10.1029/2019JD030581 PMC6988499 32025453

[15] G. Myhre , Radiative forcing due to stratospheric water vapour from CH _4_ oxidation. Geophys. Res. Lett. **34**, 2006GL027472 (2007).

[16] D. F. Hurst , Stratospheric water vapor trends over Boulder, Colorado: Analysis of the 30 year Boulder record. J. Geophys. Res. **116**, D02306 (2011).

[17] S. Kirschke , Three decades of global methane sources and sinks. Nat. Geosci. **6**, 813–823 (2013).

[18] D. F. Hurst , Closure of the total hydrogen budget of the northern extratropical lower stratosphere. J. Geophys. Res. **104**, 8191–8200 (1999).

[19] C. Dong, Q. Fu, Stratosphere-troposphere exchange of water vapor based on observations and reanalyses. Geophys. Res. Lett. **52**, e2025GL117115 (2025).

[20] R. Ueyama , Convective impact on the global lower stratospheric water vapor budget. JGR Atmos. **128**, e2022JD037135 (2023).

[21] P. K. Wang, Moisture plumes above thunderstorm anvils and their contributions to cross-tropopause transport of water vapor in midlatitudes. J. Geophys. Res. **108**, 2002JD002581 (2003).

[22] J. R. Holton , Stratosphere-troposphere exchange. Rev. Geophys. **33**, 403–439 (1995).

[23] M. De Mazière , Validation of ACE-FTS v2.2 methane profiles from the upper troposphere to the lower mesosphere. Atmos. Chem. Phys. **8**, 2421–2435 (2008).

[24] P. F. Bernath, The atmospheric chemistry experiment (ACE). J. Quant. Spectrosc. Radiat. Transfer **186**, 3–16 (2017).

[25] Y.-H. Kuo , Inversion and error estimation of GPS radio occultation data. J. Meteorol. Soc. Japan **82**, 507–531 (2004).

[26] R. A. Anthes , The COSMIC/FORMOSAT-3 mission: Early results. Bull. Amer. Meteorol. Soc. **89**, 313–334 (2008).

[27] B.-R. Wang, X.-Y. Liu, J.-K. Wang, Assessment of COSMIC radio occultation retrieval product using global radiosonde data. Atmos. Meas. Tech. **6**, 1073–1083 (2013).

[28] T. S. L’Ecuyer, N. B. Wood, T. Haladay, G. L. Stephens, P. W. Stackhouse Jr., Impact of clouds on atmospheric heating based on the R04 CloudSat fluxes and heating rates data set. J. Geophys. Res.: Atmos. **113**, D009951 (2008).

[29] D. S. Henderson, T. L’Ecuyer, G. Stephens, P. Partain, M. Sekiguchi, A multisensor perspective on the radiative impacts of clouds and aerosols. J. App. Meteorol. Climatol. **52**, 853–871 (2013), 10.1175/JAMC-D-12-025.1.

[30] M. Wang, Q. Fu, Stratosphere-troposphere exchange of air masses and ozone concentrations based on reanalyses and observations. JGR Atmos. **126**, e2021JD035159 (2021).

[31] A. Agustí-Panareda , Technical note: The CAMS greenhouse gas reanalysis from 2003 to 2020. Atmos. Chem. Phys. **23**, 3829–3859 (2023).

[32] H. Hersbach , The ERA5 global reanalysis. Q. J. R. Meteorol. Soc. **146**, 1999–2049 (2020).

[33] O. Morgenstern , Review of the global models used within phase 1 of the Chemistry-Climate Model Initiative (CCMI). Geosci. Model Dev. **10**, 639–671 (2017).

[34] D. Plummer , CCMI-2022: A new set of Chemistry-Climate Model Initiative (CCMI) community simulations to update the assessment of models and support upcoming ozone assessment activities. SPARC Newsl. **57**, 22–30 (2021).

[35] J. Liu , Change in tropospheric ozone in the recent decades and its contribution to global total ozone. J. Geophys. Res. Atmos. **127**, e2022JD037170 (2022).

[36] P. R. Colarco, CCMI-2022: refD2 Data Produced by the GEOSCCM Model at NASA-GSFC (NERC EDS Centre for Environmental Data Analysis, 2025) [Deposited 2025].

[37] C. Appenzeller, J. R. Holton, K. H. Rosenlof, Seasonal variation of mass transport across the tropopause. J. Geophys. Res. **101**, 15071–15078 (1996).

[38] M. R. Schoeberl, Extratropical stratosphere-troposphere mass exchange. J. Geophys. Res. **109**, 2004JD004525 (2004).

[39] M. A. Olsen, A. R. Douglass, T. B. Kaplan, Variability of extratropical ozone stratosphere–troposphere exchange using microwave limb sounder observations. J. Geophys. Res. Atmos. **118**, 1090–1099 (2013).

[40] M. Wang, Q. Fu, S. Solomon, B. Alexander, R. H. White, Stratosphere-troposphere exchanges of air mass and ozone concentration in the last glacial maximum. JGR Atmos. **127**, e2021JD036327 (2022).

[41] M. Wang, Q. Fu, Changes in stratosphere-troposphere exchange of air mass and ozone concentration in CCMI models from 1960 to 2099. JGR Atmos. **128**, e2023JD038487 (2023).

[42] A. Hall, Q. Fu, C. Dong, Revisiting the stratosphere-troposphere exchange of air mass and ozone based on reanalyses and observations. Atmosphere **16**, 1050 (2025).

[43] P. K. Patra , TransCom model simulations of CH_4_ and related species: Linking transport, surface flux and chemical loss with CH_4_ variability in the troposphere and lower stratosphere. Atmos. Chem. Phys. **11**, 12813–12837 (2011).

[44] D. Shindell , Simultaneously mitigating near-term climate change and improving human health and food security. Science **335**, 183–189 (2012). 22246768 10.1126/science.1210026

[45] S. Kirschke , Three decades of global methane sources and sinks. Nat. Geosci. **6**, 813–823 (2013).

[46] C. D. Boone, K. A. Walker, P. F. Bernath, “Version 3 retrievals for the atmospheric chemistry experiment fourier transform spectrometer (ACE-FTS)” in *The Atmospheric Chemistry Experiment (ACE) at 10: A Solar Occultation Anthology*, P. F. Bernath, Ed. (2013), pp. 103–127.

[47] E. R. Kursinski, G. A. Hajj, J. T. Schofield, R. P. Linfield, K. R. Hardy, Observing Earth’s atmosphere with radio occultation measurements using the Global Positioning System. J. Geophys. Res. Atmos. **102**, 23429–23465 (1997).

[48] G. A. Hajj , CHAMP and SAC-C atmospheric occultation results and intercomparisons. J. Geophys. Res. Atmos. **109**, 2003JD003909 (2004).

[49] P. Alexander, A. de la Torre, P. Llamedo, R. Hierro, Precision estimation in temperature and refractivity profiles retrieved by GPS radio occultations. J. Geophys. Res. Atmos. **119**, 8624–8638 (2014).

[50] M. J. McGill , Airborne validation of spatial properties measured by the CALIPSO lidar. J. Geophys. Res. Atmos. **112**, 2007JD008768 (2007).

[51] A. Inness , The CAMS reanalysis of atmospheric composition. Atmos. Chem. Phys. **19**, 3515–3556 (2019).

[52] R. Gelaro , The modern-era retrospective analysis for research and applications, version 2 (MERRA-2). J. Climate **30**, 5419–5454 (2017). 10.1175/JCLI-D-16-0758.1 PMC6999672 32020988

[53] World Meteorological Organization (WMO), Scientific Assessment of Ozone Depletion: 2010 (World Meteorological Organization, 2011).

[54] M. Meinshausen , The RCP greenhouse gas concentrations and their extensions from 1765 to 2300. Clim. Change **109**, 213–241 (2011).

[55] B. C. O’Neill , The Scenario Model Intercomparison Project (ScenarioMIP) for CMIP6. Geosci. Model Dev. **9**, 3461–3482 (2016). 10.5194/gmd-9-4521-2016 PMC5911933 29697697

[56] World Meteorological Organization (WMO), Scientific Assessment of Ozone Depletion: 2018 (World Meteorological Organization, 2018).

[57] P. F. Bernath, *et a*l., Global satellite-based atmospheric profiles from atmospheric chemistry experiment sciSat level 2 processed data, v5.2, 2004-2024. Federated Research Data Repository. 10.20383/103.01245. Deposited 7 April 2025.

[58] UCAR COSMIC Program, COSMIC-1 data roducts. UCAR/NCAR - COSMIC. 10.5065/ZD80-KD74. Accessed 15 January 2024.

[59] CloudSat Data Processing Center, CloudSat 2B-FLXHR-LIDAR Level 2B Radiative Fluxes and Heating Rates Data. https://www.cloudsat.cira.colostate.edu/data-products/2b-flxhr-lidar. Accessed 1 June 2024.

[60] Copernicus Atmosphere Monitoring Service, CAMS global reanalysis (EAC4) monthly averaged fields. ECMWF. 10.24381/FD75FFF2. Deposited 1 June 2025.

[61] Copernicus Climate Change Service, Complete ERA5 global atmospheric reanalysis. Copernicus Climate Change Service (C3S) Climate Data Store (CDS). 10.24381/CDS.143582CF. Deposited 1 October 2023.

[62] Global Modeling And Assimilation Office, S. Pawson, MERRA-2 tavgM_3d_rad_Np: 3d, monthly mean, time-averaged, pressure-level, assimilation, radiation diagnostics V5.12.4. NASA Goddard Earth Sciences Data and Information Services Center. 10.5067/H3YGROBVBGFJ. Accessed 1 October 2023.

[63] M. I. Hegglin, J. -F. Lamarque, V. Eyring, The IGAC/SPARC chemistry-climate model initiative phase-1 (CCMI-1) model data output. http://catalogue.ceda.ac.uk/uuid/9cc6b94df0f4469d8066d69b5df879d5. Accessed 10 January 2025.

[64] Centre for Environmental Data Analysis (CEDA), CCMI-2022 chemistry-climate model initiative, phase 2. https://catalogue.ceda.ac.uk/uuid/92dddf542adc44b5898f535be4179705. Accessed 10 October 2025.

[65] Q. Fu, C. Dong, Analysis codes and processed data for Fu and Dong (2025) PNAS paper. Zenodo. 10.5281/zenodo.17335838. Deposited 12 October 2025.

