# Supplementary material for: Global stratospheric methane loss from satellite observations
Source: Proc Natl Acad Sci U S A. 2026 Feb 9;123(7):e2529774123. doi: 10.1073/pnas.2529774123 (PMC12912900; doi:10.1073/pnas.2529774123)
Supplement: Supplementary file 1 — Appendix 01 (PDF) [file pnas.2529774123.sapp.pdf]

Supporting Information for

Global Stratospheric Methane Loss from Satellite Observations

Qiang Fu* and Cong Dong

*Corresponding author: Dr. Qiang Fu (qfu@uw.edu)

This file includes:
Tables S1 to S3
Figs. S1 to S7

Table S1. Same as Table 1, but based on CCMI-1 simulations.

	L_{OV}	L_{LM}	L_{STR}	L_{200hPa}
MMM*	21.4<br>(11.9, 30.6)	1.4<br>(0.9, 2.1)	22.8<br>(12.8, 32.7)	26.2<br>(14.7, 37.7)

* Nine CCMI-1 models are used; among them, three provide both radiative heating rates and CH_4 chemical loss.

Table S2. Same as Table 2, but based on CCMI-1 simulations.

	NH extratropics	SH extratropics	Tropics	Extratropics	Global
$F_{d,FI}^{CH_4}$	-293.0<br>(-350.2, -265.0)	-238.0<br>(-291.8, -201.9)	552.5<br>(508.0, 661.6)	-530.9<br>(-642.1, -487.2)	21.6<br>(13.3, 30.7)
$dM_{CH4,LM}/dt$	0.0<br>(-2.2, 2.4)	0.1<br>(-1.2, 1.2)	0.0	0.1<br>(-3.4, 2.0)	0.1<br>(-3.4, 2.0)
L_{LM}	0.7<br>(0.5, 1.0)	0.7<br>(0.4, 1.3)	0.0	1.4<br>(0.9, 2.1)	1.4<br>(0.9, 2.1)
$F_{trop}^{CH_4}$	-292.3<br>(-349.2, -264.6)	-237.1<br>(-290.9, -200.7)	552.5<br>(508.0, 661.6)	-529.4<br>(-640.0, -485.4)	23.1<br>(16.2, 32.5)

Table S3. Uncertainties in the CH_4 diabatic flux across the isentropic surface fitted to the tropical tropopause ($F_{d,FI}^{CH_4}$), arising from observational uncertainties in CH_4 concentrations, temperature, and diabatic heating, as well as their combined (total) uncertainty, for the NH extratropics, SH extratropics, tropics, combined extratropics, and the globe. Units are Tg/yr.

$F_{d,FI}^{CH_4}$	NH extratropics	SH extratropics	Tropics	Extratropics	Global
CH_4	42.1	40.7	87.6	82.8	4.8
T	1.9	1.1	3.0	3.0	0.1
Diabatic heating	37.8	59.4	99.6	97.2	5.7
Total	56.6	72.0	132.7	127.7	7.5

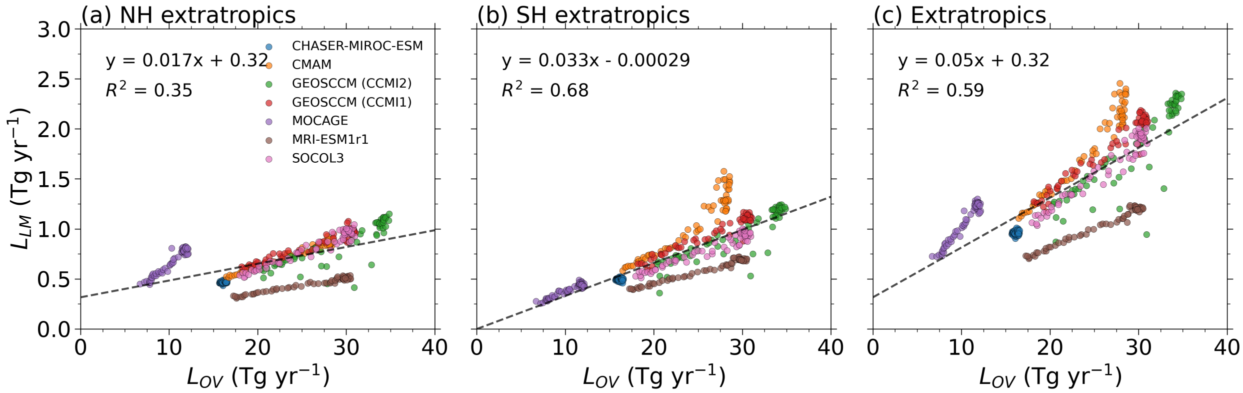

Figure S1. Annual-mean CH_4 chemical loss in the lowermost stratosphere (L_{LM}) for (a) NH extratropics, (b) SH extratropics and (c) combined extratropics versus CH_4 chemical loss in the overworld (L_{OV}), based on CCMI model simulations. Different colors denote individual models, and the black dashed line represents the linear regression fit. The regression equation and R^2 value are shown in the upper-left corner. Only one CCMI-2 model provides CH_4 chemical loss, compared with six models from CCMI-1.

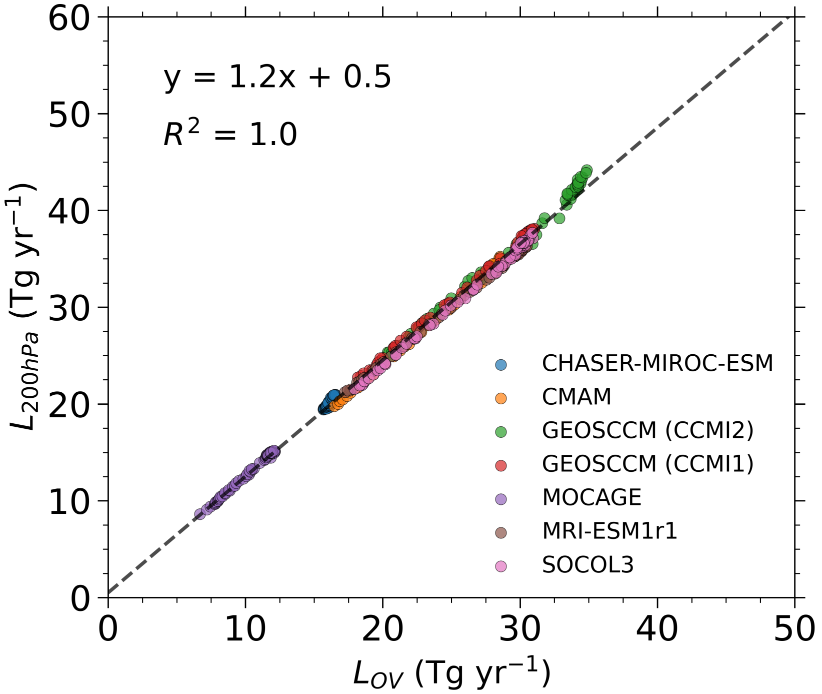

Figure S2. Annual-mean CH_4 chemical loss above 200 hPa (L_{200hPa}) versus CH_4 chemical loss in the overworld (L_{OV}), based on CCMI model simulations. Different colors denote individual models, and the black dashed line represents the linear regression fit. The regression equation and R^2 value are shown in the upper-left corner. Only one CCMI-2 model provides CH_4 chemical loss, compared with six models from CCMI-1.

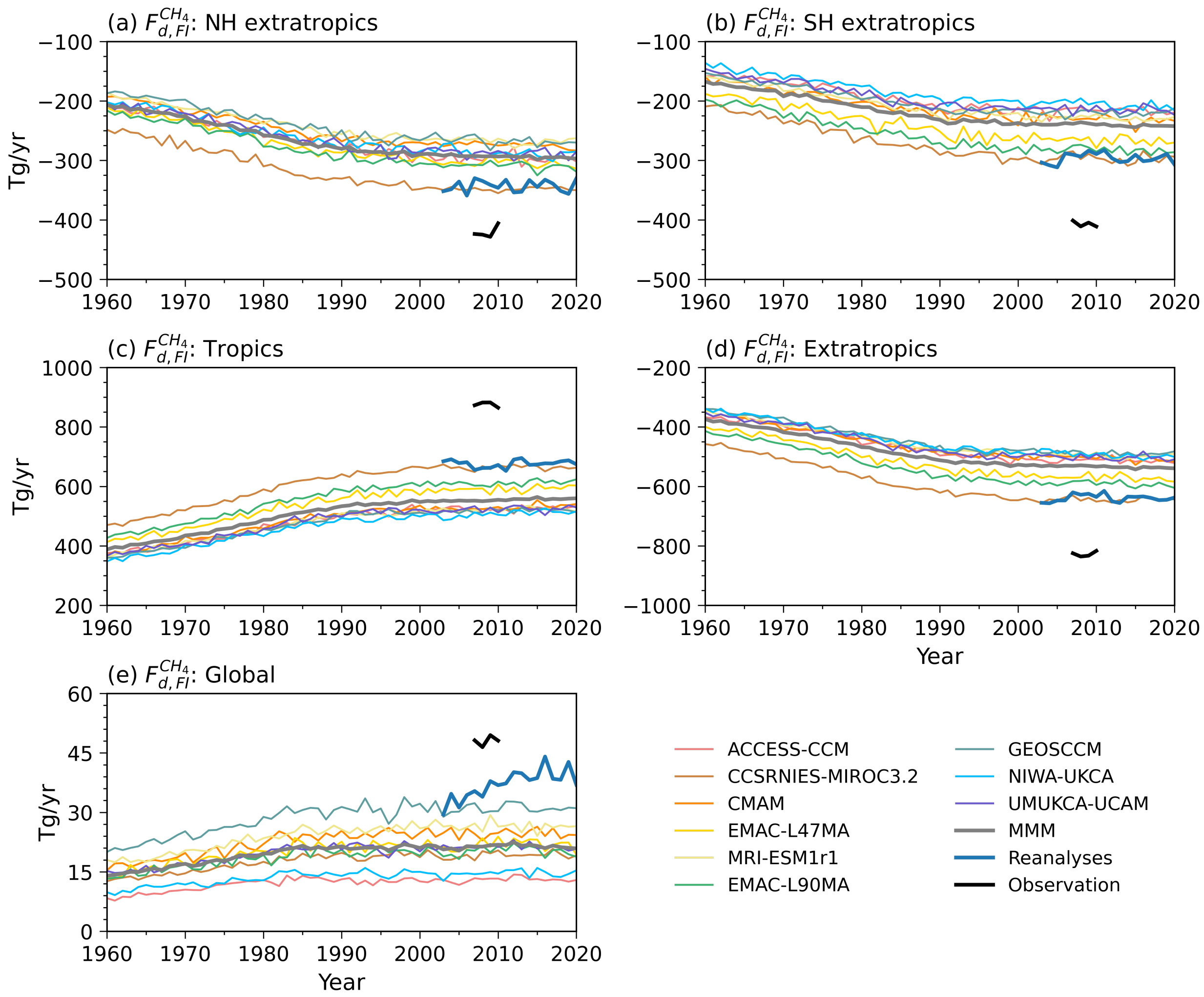

Figure S3. Same as Fig. 3, but using CCMI-1 model simulations.

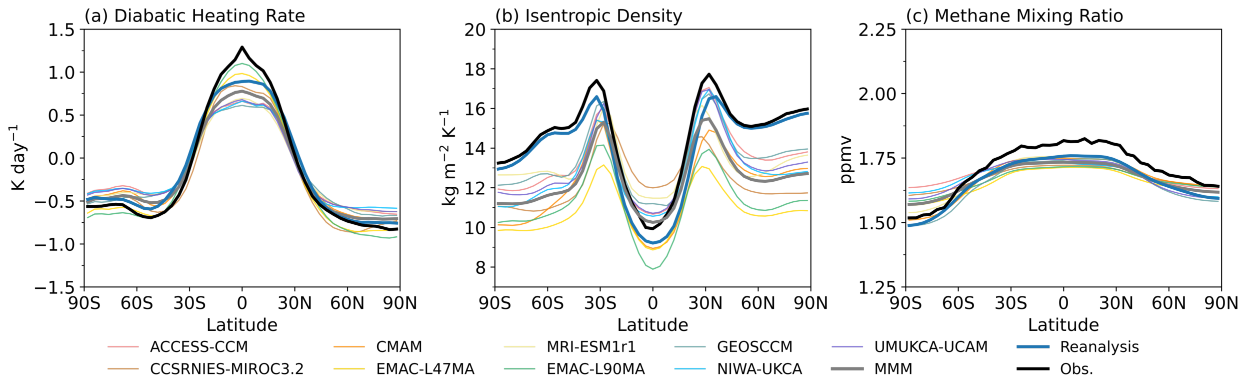

Figure S4. Same as Fig. 4, but using CCMI-1 model simulations.

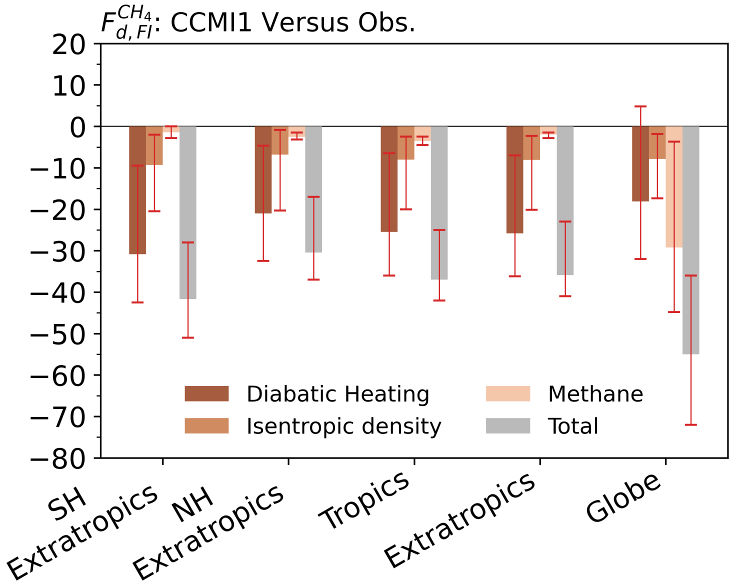

Figure S5. Same as Fig. 5b, but using CCMI-1 model simulations.

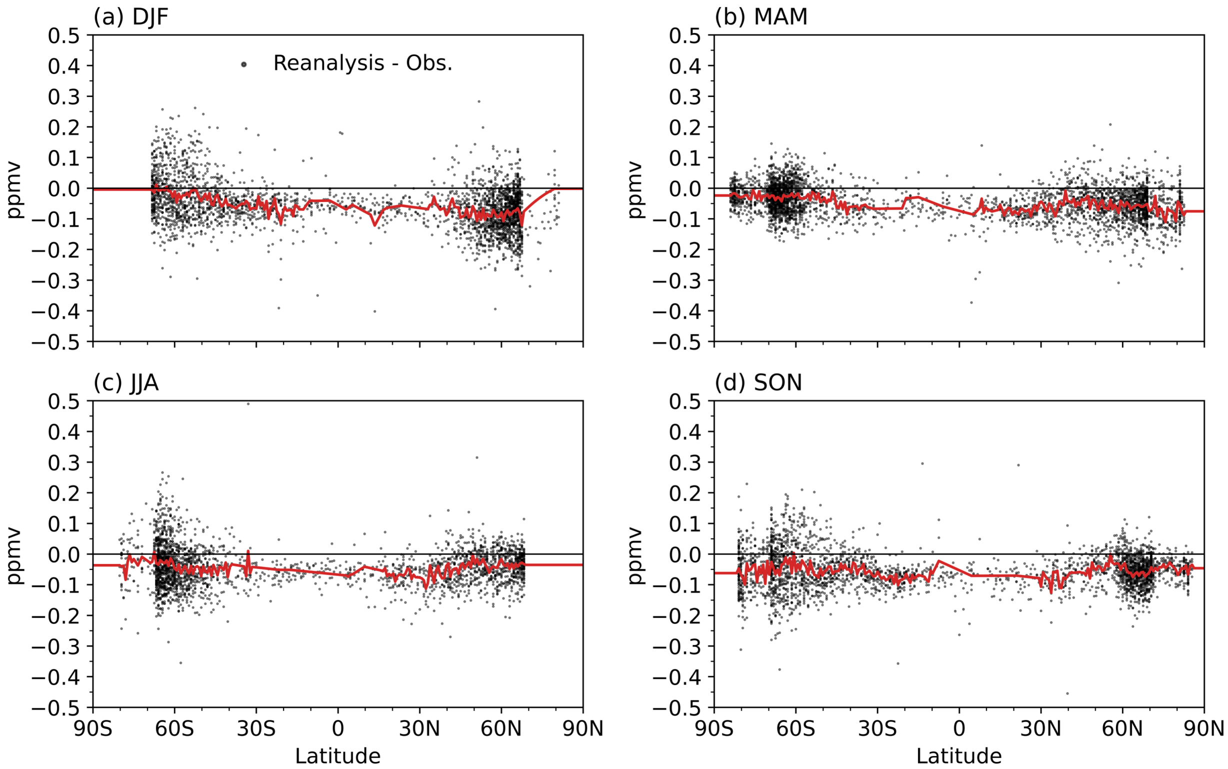

Figure S6. Reanalysis-observation CH_4 differences as a function of latitude for four seasons over 2007-2010, evaluated at the isentropic surface fitted to the tropical tropopause as an example. Each black dot represents the difference between the reanalysis and observations at a grid cell for a given month with available CH_4 observations. The red lines show the seasonal zonal-mean climatology of the reanalysis–observation differences, with data gap filled by interpolation at mid- and low-latitudes and by the nearest-neighbor values at high-latitudes. The black lines indicate the zero reference line.

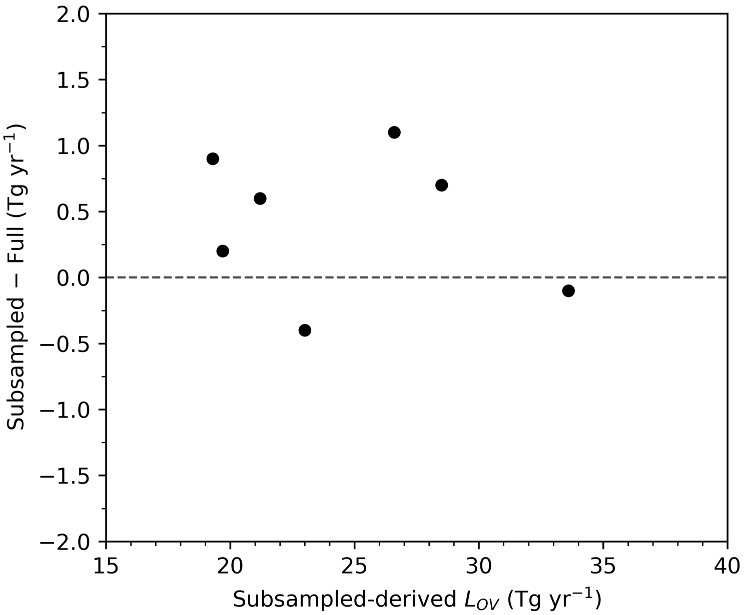

Figure S7. Differences between subsampled CH_4-derived and full CH_4-derived CH_4 chemical loss in the overworld (L_{OV}), averaged over 2007-2010. The subsampled CH_4-derived L_{OV} (x-axis) were obtained using CCMI-2 model CH_4 fields extracted at the grid cells corresponding to ACE-FTS observation locations for each month, with missing data filled following the method described in the Materials and Methods. The full CH_4-derived fluxes represent values calculated from the complete model CH_4 fields without subsampling. Results are based on seven CCMI-2 model. The black dashed line indicates the zero reference line. The mean difference is 0.4 Tg/yr.
